# Work engagement and associated factors among dental nurses in China

**DOI:** 10.1186/s12903-021-01766-y

**Published:** 2021-08-16

**Authors:** Yujing Wang, Yuqin Gao, Yang Xun

**Affiliations:** Department of Nursing, School and Hospital of Stomatology, China Medical University, Liaoning Provincial Key Laboratory of Oral Diseases, Shenyang, 110002 People’s Republic of China

**Keywords:** Work engagement, Job stress, Perceived social support, Subjective well-being, Psychological flexibility, Nurse

## Abstract

**Background:**

Work engagement is affected by many factors. The level of work engagement among dental nurses is unknown.

**Methods:**

A cross-sectional questionnaire survey was conducted among 215 dental nurses. The Utrecht Work Engagement Scale, Chinese Nurse Stressors Scale, Work-related Acceptance and Action Questionnaire, Multi-dimensional Scale of Perceived Social Support, and General Well-Being Schedule were applied to measure Chinese nurses’ work engagement, job stress, psychological flexibility, perceived social support and subjective well-being, respectively. Univariate analysis was used to identify the relationships of work engagement with demographic and psychological characteristics. Hierarchical linear regression analysis was applied to test the variance in work engagement accounted for by factors related to work engagement in the univariate analysis.

**Results:**

The level of work engagement among Chinese dental nurses was moderate or above. Work engagement was positively associated with perceived social support, psychological flexibility and subjective well-being but negatively correlated with job stress. The hierarchical regression analysis showed that age, job stress, psychological flexibility and subjective well-being were significantly correlated with work engagement, though perceived social support was not, all of those psychological variables together explained 34.7% of the variance in work engagement.

**Conclusions:**

Dental nurses in China had an acceptable level of work engagement in terms of vigour, dedication and absorption. Increased job stress resulted in lower work engagement. Nurses who had higher levels of perceived social support, psychological flexibility and subjective well-being also had higher work engagement. It is necessary to understand the job stress of nurses, strengthen nurses’ social support, relieve nurses’ job stress, improve nurses’ psychological flexibility and subjective well-being, which will improve nurses’ work engagement levels.

## Background

Good oral health is a starting point for people’s general health and well-being [1]. As a branch of clinical medicine, stomatology has its own distinctive characteristics. One of these characteristics is that stomatologists cannot complete diagnosis and treatment alone; they must have relevant auxiliary personnel to cooperate and carry out four-handed operations to complete treatment smoothly and with high quality. Auxiliary personnel are dental hygienists or dental therapists in America, England, Australia, and Japan [2, 3]. In China, this role is performed by dental nurses. In addition to cooperating with stomatologists to complete treatment, they undertake the tasks of reception, triage services, health education, medical guidance, and oral health care [4–6]. Currently, the training of dental nurses in China is still in the exploratory stage [7, 8]. It mainly includes in-service training and continuing education and lacks systematic professional education. Education between different nursing majors among nursing colleges in China hasn’t been distinguished. Nurses receive different professional knowledge and skills training in their work units after graduation. All nurses have obtained the professional qualification certificate through a unified examination. However, the profession of dental nurses, namely dental assistants in other countries, has been set up as a special major in colleges and students are engaged in the work of dental assistants after graduation, dental assistants have received systematic education of oral nursing in colleges. While education among dental nurses in China mainly relies on clinical continuing education. Lacking of teaching experience and shorter time of training will lead to a different teaching effect and learning effect, which may affect the work engagement level of dental nurses. These factors hinder the quality requirements of dental nurses, especially in work engagement.

Work engagement is a positive, substantial, work-related mental state that includes vigour (i.e., energy), dedication (i.e., involvement and significance) and absorption (i.e., concentration) [9]. Work engagement has been found to have a direct impact on the quality of care [10, 11]. In addition, it has been found that engaged employees have less job stress and depression than nonengaged employees [12] and that a high level of work engagement can enhance nurses' job performance, job satisfaction, and emotional health and reduce turnover intention [13]. Since work engagement is important to both organizations and individuals, it is vital to investigate the factors associated with work engagement among dental nurses.

Several factors have been reported to be related to work engagement. The job demands-resources (JD-R) model is used to explain the factors related to work engagement. According to the JD-R [14], factors that affect work engagement include work-related factors and individual factors. Work-related factors are categorized into four domains: work stressors (job aspects that require substantive mental and physical effort), work resources (job aspects that stimulate personal development and help to achieve work goals), work psychosocial emotions (employees’ emotional and mental outcomes from work) and work outcomes (work performance indicators). Individual factors are categorized into three domains: demographics, individual health (mental, psychological and physical health) and personal factors (factors outside of work). Several factors have been reported to be related to the level of work engagement. Work engagement is a result of personal learning (i.e., sensibility, reflection) and the work environment [5], with the work environment possibly playing a more important role in work engagement. Some studies have concluded that job stress is positively associated with work engagement [15], while other studies have found opposite results, i.e., that job stress is negatively associated with work engagement [16, 17]. Psychological flexibility [18], perceived social support [19] and subjective well-being [20] have also been found to be associated with work engagement. However, little information about work engagement among dental nurses is currently available.

Researchers have increasingly recognized the value of work engagement, and exploring the relevant factors of work engagement is crucial. While these variables have effects on work engagement, they are not mutually exclusive, and their integrated effects on nurses remain unknown, especially among dental nurses in China. China faces the challenges of population aging and “three-child” policy, women have to work as well as taking care of their families. Lacking of energy may lead to the decrease of nurses' work engagement. Dental nurses work closely with doctors, low work engagement may lead to the decline of nursing quality and patients' satisfaction. The true demand for dental service in China was relatively low and not seen as critical. Compared with other professional nursing, the social status and income of dental nurses in China are not as high as those of nurses in general hospitals, which may affect nurses’ work engagement. While there are few studies on work engagement among dental nurses in China, therefore, to investigate the work engagement among dental nurses and its associated factors is helpful for nursing managers to provide effective organizational support and improve work engagement of nurses. According to the JD-R model, work-related factors such as job stress, individual factors such as demographics and psychological flexibility, subjective well-being and social support related to both work and individual factors were included in this study. We propose that job stress is negatively associated with work engagement and that psychological flexibility, perceived social support, and subjective well-being are positively associated with work engagement. We test these hypotheses in the current study. The aim of the current study is to explore the level of work engagement of dental nurses in China and to identify key factors that potentially influence work engagement.

## Methods

### Design

Declaration of Helsinki was used to guide this study. A cross-sectional questionnaire survey was conducted among dental nurses. All procedures were performed in accordance with relevant guidelines. All scales were authorized for use.

### Sample and setting

The research was conducted at the School and Hospital of Stomatology, China Medical University, in December 2020. This is the largest hospital of stomatology in Northeast China and has more than 245 dental chairs and 110 beds. The study was approved by the Committee on Human Experimentation of China Medical University (2020–22). A convenience sample of nurses from the School and Hospital of Stomatology, China Medical University, participated in the survey. The sample size [21–23] was calculated as follows: $n = \frac{{Z_{\alpha }^{2} \sigma ^{2} }}{{\delta ^{2} }}$. The parameters were α = 0.05, Zα = 1.96, σ = 14.12, δ = 2. n = 1.96^2^ * 14.12^2^/2^2^ = 191.48. The σ represents standard deviation. The value of σ = 14.12 was calculated from a pre-experiment conducted among 30 nurses. The δ represents the tolerance error, the smaller the δ is, the larger the sample size and accuracy are. Generally, the tolerance error can be set by 0.25 or 0.50 times of standard deviation. In this study, σ = 14.12, the value of δ can be 3.3–7.06, while in order to improve the accuracy of the results, we selected 2 in the study. Given the possibility of invalid questionnaires, the sample size was increased by 10–20%, and the final sample size was 212–231. Before the study, an informed consent form was distributed to and collected from nurses. Nurses were included if they (1) had > 1 working year, (2) had obtained a nurse qualification certificate, and (3) provided informed consent. Nurses were excluded if they (1) were on maternity leave, (2) had > 3 months of sick leave, or (3) were not engaged in nursing. A total of 254 nurses met the inclusion criteria, and questionnaires were sent via an online QR code. The questionnaire response time was 20 min. Nurses participated in the survey through the online QR. Data collection took place over 3 days (29–31 December 2020). All questions were set to be required to answer, nurses who missed any questions could not submit. Of the 254 nurses who were sent questionnaires, 84.6% responded and completed the questionnaires without missing data. The final sample size was 215 nurses.

## Measurement

### Work engagement

The 9-item Utrecht Work Engagement Scale (UWES) [24] was used to evaluate nurses’ work engagement. The UWES includes three dimensions: vigour (3 items), dedication (3 items), and absorption (3 items). Each item is rated on a 7-point scale, where 0 is “never” and 6 is “always”. The total score is computed by summing the score of the three dimensions. The ratio of the total score to the item number is the total average score, and the ratio of each dimension score to the item number is the average dimension score. A higher score indicates a higher level of work engagement. The UWES has been proven to have satisfactory reliability and validity in China [25]. The Cronbach’s alpha in this study was 0.948.

### Job stress

We used the Chinese Nursing Stress Scale (CNSS) to measure dental nurses’ job stress [26]. The CNSS has been proven to be useful and reliable for evaluating the job stress of nurses and has been widely used in China [27]. It contains 35 items across five dimensions: stress from nursing practice and care (7 items), stress from workload and time allocation (5 items), stress from the work environment and resources (3 items), stress from patient care (11 items), and stress from management and relationships (9 items). Each item is rated on a 4-point scale ranging from 1 (“never”) to 4 (“almost every day”). The total score ranges from 35 to 140. A higher score indicates higher job stress. The Cronbach’s alpha was 0.960 in this study.

### Psychological flexibility

We used the Work-related Acceptance and Action Questionnaire (WAAQ) [28] to measure nurses’ psychological flexibility in occupational settings. Xu et al. translated the WAAQ to Chinese in 2018, and it has been proven to be useful and reliable, with a Cronbach’s α of 0.920 [29]. The WAAQ includes 7 items rated on a 7-Likert scale from 1 (“never”) to 7 (“always”). The total score of the WAAQ ranges from 7 to 49; a higher WAAQ indicates better psychological flexibility, better job acceptance, and greater work activity. The Cronbach’s alpha in this study was 0.949.

### Perceived social support

Zimet et al. [30] developed the Multidimensional Scale of Perceived Social Support (MSPSS) in 1988. The MSPSS includes 12 items, with each item rated on a 7-point Likert-type scale, where 1 indicates “very strongly disagree” and 7 indicates “very strongly agree”. The total score of the MSPSS ranges from 12 to 84. A higher score indicates better social support and higher satisfaction. The scale contains 3 dimensions: family support (4 items), friend support (4 items) and other support (e.g., relatives and colleagues). The scale emphasizes individual understanding and feelings regarding perceived social support. The Chinese version of the MSPSS has been widely used in China and has been proven to be a good measure to evaluate an individual’s perceived social support [31]. The Cronbach’s alpha in this study was 0.975.

### Subjective well-being

The General Well-Being Schedule (GWBS) was used to assess nurses’ subjective well-being. Duan translated and revised the schedule into Chinese in 1996 [32]. Duan summarized and analysed international research on happiness and formulated a definition of happiness based on three aspects: others' evaluation of individuals, individuals’ own emotional experience and self-evaluation. The Chinese version of the GWBS contains 18 items across 6 dimensions: general health (2 items), vigour (3 items), positive well-being (3 items), depression (5 items), self-control (3 items), and anxiety (2 items). Among the 18 items, 4 items are rated on a 5-point Likert-type scale, 10 items are rated on a 6-point Likert-type scale, and the other 4 items are rated on a 0–10 rating scale. The total GWBS score ranges from 14 to 120. A higher score reflects a higher level of general well-being. The Chinese version of the GWBS has been widely applied in China and has proven to be useful to measure individuals’ subjective well-being [33, 34]. The Cronbach’s alpha in this study was 0.881.

### Statistical analyses

The demographic data are expressed as frequencies and percentages. Shapiro–Wilk test was used to test the normal distribution of continuous variables. All continuous variables were non-normal distributed except GWBS. Psychological characteristics were analysed with descriptive statistics. Mann–Whitney U Test and Kruskal–Wallis H Test were used to compare the work engagement of individuals with different demographic variables. Spearman's Rank-Order Correlation were used to analyse the relationships of work engagement with job stress, psychological flexibility, perceived social support and subjective well-being. The relationships of the demographic data, job stress, psychological flexibility, perceived social support and subjective well-being to work engagement identified in the Mann–Whitney U Test and Kruskal–Wallis H Test were further examined through hierarchical linear regression. After transformation of normality(square root transformation), demographic data related to work engagement were entered in step one, and psychological variables such as job stress, psychological flexibility, perceived social support and subjective well-being were entered in step two. VIF was used to test whether there was multicollinerarity among the variables. VIF < 5 was considered that there was no multicollinerarity.

## Results

### Demographic characteristics and level of work engagement

The characteristics of the nurses and the level of work engagement among different subgroups are displayed in Table 1. The work engagement scores differed among nurses of different ages, professional titles and engagement in exercise (*p* < 0.05).

**Table 1:** Demographic characteristics and level of work engagement among nurses (N = 215)

Characteristics	N (%)	UWES		
		M (IQR)	Z/X^2^	*P*
*Gender*			− 0.166	0.868
Male	2 (0.9)	40.00 (0)		
Female	213 (99.1)	36.00 (16)		
*Age*			12.932	0.012*
< 30 years	106 (49.3)	36.00 (16)		
31–35 years	75 (34.9)	36.00 (13)		
36–40 years	22 (10.2)	37.00 (15)		
41–45 years	10 (4.7)	52.00 (9)		
> 46 years	2 (0.9)	36.50 (0)		
*Marital status*			− 0.339	0.735
unmarried or other	76 (35.3)	37.00 (16)		
married	139 (64.7)	36.00 (16)		
*Highest degree*			0.449	0.503
junior college	33 (15.3)	36.00 (23)		
undergraduate	178 (82.8)	36.00 (15)		
master’s	4 (1.9)	26.00 (24)		
*Exercise*			− 2.367	0.018*
Yes	101 (47.0)	35.00 (14)		
No	114 (53.0)	39.00 (17)		
*Post*			− 0.947	0.343
nurse	201 (93.5)	36.00 (16)		
head nurse	14 (6.5)	39.50 (16)		
*Shift type*			− 0.731	0.465
Night shift	35 (16.3)	34.00 (16)		
Day shift	180 (83.7)	36.00 (16)		
*Professional titles*			10.001	0.007*
nurse	39 (18.1)	37.00 (16)		
senior nurse	152 (70.7)	35.00 (14)		
chief nurse	24 (11.2)	45.50 (19)		
*Department*			− 0.694	0.488
Outpatient	154 (71.6)	36.00 (17)		
Inpatient	61 (28.4)	37.00 (14)		
*Working years*			9.161	0.057
1–5 years	48 (22.3)	36.00 (16)		
6–10 years	100 (46.5)	35.00 (13)		
11–15 years	42 (19.5)	39.00 (20)		
16–20 years	15 (7.0)	37.00 (13)		
> 20 years	10 (4.7)	47.50 (17)		
*Household income*			4.742	0.315
< 10 thousand	78 (36.3)	36.50 (16)		
11–15 thousand	65 (30.2)	36.00 (15)		
16–20 thousand	44 (20.5)	35.00 (16)		
20–30 thousand	18 (8.4)	40.50 (22)		
> 30 thousand	10 (4.7)	35.50 (18)		

Mann–Whitney U Test and Kruskal–Wallis H Test were used in the table

UWES = Utrecht Work Engagement Scale

*p < 0.05

### UWES and average subscale scores in Chinese dental nurses

The total work engagement score and dimension scores for Chinese nurses are shown in Table 2;

**Table 2:** UWES and average subscale scores for Chinese dental nurses (N = 215)

*UWES*	Item	Min	Max	M (IQR)
Total	9	6	54	36 (16)
Vigour	3	3	18	12 (5)
Dedication	3	1	18	12 (6)
Absorption	3	2	18	12 (6)

UWES Utrecht work engagement scale

### Analysis of the relationships of work engagement with psychological characteristics

The analysis of the relationships of work engagement with psychological characteristics is displayed in Table 3. The psychological variables, including the WAAQ score (ρ = 0.563, *p* < 0.001), MSPSS score (ρ = 0.427, *p* < 0.001) and GWBS score (ρ = 0.418, *p* < 0.001), were found to be positively associated with work engagement, while the CNSS score (ρ = −0.422, *p* < 0.001) was negatively associated with work engagement.

**Table 3:** The relationships between work engagement and psychological characteristics (N = 215)

	UWES	CNSS	WAAQ	MSPSS	Median/Mean	IQR/SD
UWES					36	16
CNSS ρ	− 0.422***				65	19
WAAQ ρ	0.563***	− 0.413***			38	13
MSPSS ρ	0.427***	− 0.408***	0.588***		72	18
GWBS ρ	0.418***	− 0.548***	0.392***	0.473***	68.87	11.85

Spearman's Rank-Order Correlation was used in the table

UWES = Utrecht work engagement scale; CNSS = Chinese nurse stressors scale; WAAQ = work-related acceptance and action questionnaire; MSPSS = multi-dimensional scale of perceived social support; GWBS = general well-being schedule

***p < 0.001 (two-tailed)

### Hierarchical linear regression analysis of work engagement

A hierarchical linear regression analysis was performed to test the variance in work engagement accounted for by factors related to work engagement in univariate analysis. Demographic data, job stress, resilience, social support and subjective well-being, which were found to be related to job engagement, were entered into the regression equation. Because age and professional title would be strongly correlated each other(r = 0.545, *p* < 0.001), the growth of age will lead to the promotion of professional title, so the two variables should not be added in the same regression model. In step one, the demographic data (age and exercise) related to work engagement were entered, and then psychological variables such as job stress, psychological flexibility, social support and subjective well-being were entered into the regression in the second step. 40–45 years and exercise were significant in step one before the addition of job stress, resilience, social support and subjective well-being variables; after these variables were added in step 2, age, job stress, psychological flexibility and subjective well-being were significantly correlated with work engagement, while perceived social support was not significant. Job stress, psychological flexibility, perceived social support and subjective well-being explained 34.7% of the variance in work engagement. The results of the hierarchical linear regression analysis are presented in Table 4.

**Table 4:** Hierarchical linear regression analysis of work engagement (N = 215)

WE	Step1 beta	VIF	P	Step 2 beta	VIF	P
Constant			0.000			
Age Reference: < 30 years						
31–35 years	0.056	1.112	0.429	0.077	1.127	0.179
36–40 years	0.031	1.084	0.659	0.082	1.131	0.152
40–45 years	0.251	1.043	0.001**	0.181	1.064	0.001**
> 46 years	0.014	1.009	0.832	0.021	1.020	0.695
CNSS				− 0.237	1.569	0.000***
WAAQ				0.326	1.209	0.000***
GWBS				0.201	1.514	0.003**
F	3.369			20.334		
P	0.011*			0.000***		
R^2^	0.060			0.407		
AdjR^2^	0.042			0.387		
AdjR^2^-change				0.347		

UWES = Utrecht work engagement scale; CNSS = Chinese nurse stressors scale; WAAQ = work-related acceptance and action questionnaire; MSPSS = multi-dimensional scale of perceived social support; GWBS = general well-being schedule; VIF = variance inflation factor; AdjR^2^ = adjusted R^2^

*p < 0.05; **p < 0.01; ***p < 0.001 (two-tailed)

## Discussion

Work engagement is influenced by many factors, such as family [35], personality [36], exercise [37], and diet [38]. A Portuguese study on work engagement among 3887 rescue workers (50% nurses, 39% firefighters and 11% policemen) showed that firefighters had the highest work engagement, while nurses had the lowest work engagement [39]. This result may be related to the external needs of nurses, the lack of manpower and resources, and conflict with or aggression by patients or their families [40]. A high level of work engagement can enhance nurses' job performance, job satisfaction, and emotional health and reduce turnover intention [8] and has a positive impact on nurses' attitudes towards patients [41]. A survey [42] among 1330 nurses in 10 general hospitals (> 500 beds) indicated that the work engagement scores among nurses in vigour (3.21), dedication (3.44) and absorption (2.73) were lower than those for 860 employees from Serbia (vigour, 3.66; dedication, 4.04; absorption, 4.23) [43]. This is likely due to the more complex occupational environment and job stress among health workers than people in other professions. The level of work engagement in Chinese dental nurses in this study was moderate or above, higher than those in general hospitals in China [35] and higher than those reported in an international study [17] conducted in 10 different countries involving different professions (e.g., social work, blue-collar, health care, teaching, police), and Simpson’s study, which was conducted among 479 registered nurses employed in six hospitals and 16 medical and/or surgical units located in one midwestern state of United States [44]. Work engagement is associated with many factors, and profession may be one of them. Stomatological hospitals in China are composed of outpatients and wards, of which outpatients account for the majority. The main content of dental nurses’ work is cooperating with doctors. They seldom need to care for patients by themselves as nurses do in general hospitals; dental nurses may therefore have lower job stress and workload, which may explain the result.

Work engagement was positively associated with perceived social support, psychological flexibility and subjective well-being but negatively correlated with job stress. We can conclude that higher perceived social support, psychological flexibility and subjective well-being result in higher work engagement. The higher job stress is, the lower work engagement is. A survey of 726 employees in Finland showed that physical and mental health factors were positively correlated with work engagement, while psychosocial risk factors (i.e., anxiety, stress) were negatively correlated with work engagement [30]. Nurses who regularly participated in exercise had higher work engagement than others in this study. Physical exercise is beneficial to physical and mental health; it can relieve pressure and help nurses engage in vigorous work. Professional title and age were the influencing factors of work engagement. Nurses with higher professional titles also had higher work engagement than nurses with lower professional titles. Nurses with high professional titles had worked for a longer time and had more work experience and stable family relationships, so they had higher work engagement. Nurses with lower professional titles often had shorter working years, a lack of experience and the pressures of marriage, birth and professional title examinations, which resulted in lower work engagement. Nurses aged 40–45 had higher work engagement than others. They may have had children who could care for themselves and fewer family distractions and therefore had higher work engagement. Nurses under the age of 40 often had few children who needed care, while nurses over 46 years old often faced the pressure of their children going to college and supporting elderly parents, so their level of work engagement was lower than that of nurses aged 40–45. Nursing managers should strengthen the organizational culture and provide more care and support to nurses with lower professional titles, younger age and older age.

In the European Working Conditions Survey (EWCS 2000), job stress was found to be the second most common work-related health problem [45]. The overall pressure of nurses has a negative impact on mental health [46, 47]. Job stress among nurses reduced patient-perceived reliability and assurance, which meant that patient-perceived nursing quality declined [48]. Higher job stress levels have many negative effects, such as reduced satisfaction, burnout, turnover and poor sleep quality. Although Keykaleh et al. [49] asserted that nurses’ job stress was not correlated with patients’ safety, Rainbow et al. suggested that job stress could lead to a decline in nurses' performance and increase the safety risk of patients [50]. Although a study conducted among 9134 employees in Japan concluded that job stress was positively associated with work engagement [10], the opposite results were found in this study: job stress was negatively associated (β = −0.194, *p* < 0.01) with work engagement, similar to other studies among nurses [11, 12]. Higher job stress may result in lower work engagement. The different results may be due to different regions, cultures and occupations. Health care workers have more psychological and physical stress than other professionals [39]. Different professions may have different levels of stress. Responding to the needs of patients quickly is the duty of health care workers. Complicated medical knowledge and skills are required daily, and any mistakes may be harmful to patients’ lives, all of which lead to higher psychological and physical stress in health care workers.

Psychological flexibility can be defined as the ability to act according to goals and values in the context of disturbing psychological experience [51]. Employees who exhibit better psychological flexibility have been found to be more open when experiencing frustration and difficulties at work and more adaptive to changes at work [38]. A higher level of psychological flexibility was also shown to be associated with greater work engagement and satisfaction [13]. A study of work engagement in 124 residents and 69 experts in five hospitals in the Netherlands reported that residents’ work engagement was associated with psychological flexibility, while experts’ work engagement was associated with colleague support [52], indicating that work engagement differs among different individuals and be affected by many factors. The hierarchical regression analysis in this study showed a significant positive correlation between work engagement and psychological flexibility, which was similar to the findings of other studies [52].

Subjective well-being refers to happiness or satisfaction, which is related to good social relations, work performance and creativity [53]. People feel happiness when they feel more pleasant emotions than unpleasant emotions. Nurses' subjective well-being is very important for both individuals and organizations as it helps increase job stability and satisfaction [54]. In many studies on subjective well-being, work engagement was found to be an intermediary factor [55], which means that an increasing level of work engagement could indirectly improve an individual’s subjective well-being. Subjective well-being was found to be moderately related to work engagement among Polish individuals [56]. A study conducted with a sample of 319 full-time in-service kindergarten teachers concluded that subjective well-being had a positive association with work engagement [15]. The hierarchical regression analysis in this study showed a significant positive correlation between work engagement and subjective well-being, similar to other studies [57]. Certain levels of work engagement can enhance subjective well-being, while workaholism is negatively associated with subjective well-being [58].

A significant positive correlation was reported between college teachers' work engagement and students' social support [14]. Providing more support at work can help female nurses achieve a balance between family and work and increase work engagement [59]. Providing independent and diverse development opportunities and social support resources can increase employees' work engagement [60]. Although perceived social support was positively correlated with work engagement in the univariate analysis, it was not positively associated with work engagement in the hierarchical regression analysis. This inconsistency may be due to psychological capital acting as a mediator between perceived social support and work engagement [61]. The findings demonstrated an inverted U-shaped curve relationship between enterprises' social support and employees' work engagement. Since perceived social support is significantly related to UWES, CNSS, WAAQ and GWBS, perceived social support may not be independently related to work engagement.

The strengths of this study include our identification of the level of work engagement among dental nurses in China for the first time. We analysed the influencing factors of work engagement and explored the influence of multiple psychological factors on work engagement. Limitations of the study include our focus only on the associations of work engagement with psychological flexibility, social support, subjective well-being and job stress; other factors that might influence work engagement were not included. Although this study was conducted in the largest stomatological hospital in Northeast China, which is influential in China, a larger and multicentre sample is still needed to improve the representativeness of the findings.

## Conclusion

Dental nurses in China had an acceptable level of work engagement in terms of vigour, dedication and absorption. Work engagement was positively associated with perceived social support, psychological flexibility and subjective well-being but negatively correlated with job stress, which implies that increased job stress results in lower work engagement. Nurses who had higher levels of psychological flexibility and subjective well-being also had higher work engagement. Job stress, psychological flexibility and subjective well-being were significantly correlated with work engagement, though perceived social support was not, all of those psychological variables together explained 34.7% of the variance in work engagement.

### Clinical implications

The results of this study suggest that nursing administration should target efforts to relieve nurses’ job stress, improve nurses’ levels of subjective well-being, psychological flexibility, enhance nurses’ professional status and social support, thereby improving nurses’ work engagement.

## Data Availability

Data and materials are available from the corresponding author upon reasonable request.

## Abbreviations

UWES: Utrecht work engagement scale
CNSS: Chinese nurse stressors scale
WAAQ: Work-related acceptance and action questionnaire
MSPSS: Multi-dimensional scale of perceived social support
GWBS: General well-being schedule
